# Spatial epidemiology and climatic predictors of paediatric dengue infections captured via sentinel site surveillance, Phnom Penh Cambodia 2011–2012

**DOI:** 10.1186/1471-2458-14-658

**Published:** 2014-06-28

**Authors:** Andrew A Lover, Philippe Buchy, Anne Rachline, Duch Moniboth, Rekol Huy, Chour Y Meng, Yee Sin Leo, Kdan Yuvatha, Ung Sophal, Ngan Chantha, Bunthin Y, Veasna Duong, Sophie Goyet, Jeremy L Brett, Arnaud Tarantola, Philippe Cavailler

**Affiliations:** 1 Infectious Diseases Programme, Saw Swee Hock School of Public Health, National University of Singapore, MD3, 16 Medical Drive, Singapore 117597, Singapore; 2 Virology Unit, Institut Pasteur in Cambodia, Phnom Penh, Cambodia; 3 Formerly- Regional Emerging Diseases Intervention (REDI) Centre, Singapore, Singapore; 4 Infectious Diseases Unit, National Paediatric Hospital, Phnom Penh, Cambodia; 5 National Dengue Control Program (NDCP), National Center for Parasitological, Entomology and Malaria Control, Ministry of Health, Phnom Penh, Cambodia; 6 Ministry of Health, Phnom Penh, Cambodia; 7 Tan Tock Seng Hospital, Centers for Disease Control, Singapore, Singapore; 8 Emergency Unit, National Paediatric Hospital, Phnom Penh, Cambodia; 9 Medical Affairs Department, Sanofi Pasteur, Singapore Regional Office, Singapore, Singapore; 10 Epidemiology Unit, Institut Pasteur in Cambodia, Phnom Penh, Cambodia; 11 Médecins Sans Frontières, Operational Centre, Geneva, Switzerland

## Abstract

**Background:**

Dengue is a major contributor to morbidity in children aged twelve and below throughout Cambodia; the 2012 epidemic season was the most severe in the country since 2007, with more than 42,000 reported (suspect or confirmed) cases.

**Methods:**

We report basic epidemiological characteristics in a series of 701 patients at the National Paediatric Hospital in Cambodia, recruited during a prospective clinical study (2011–2012). To more fully explore this cohort, we examined climatic factors using multivariate negative binomial models and spatial clustering of cases using spatial scan statistics to place the clinical study within a larger epidemiological framework.

**Results:**

We identify statistically significant spatial clusters at the urban village scale, and find that the key climatic predictors of increasing cases are weekly minimum temperature, median relative humidity, but find a negative association with rainfall maximum, all at lag times of 1–6 weeks, with significant effects extending to 10 weeks.

**Conclusions:**

Our results identify clustering of infections at the neighbourhood scale, suggesting points for targeted interventions, and we find that the complex interactions of vectors and climatic conditions in this setting may be best captured by rising minimum temperature, and median (as opposed to mean) relative humidity, with complex and limited effects from rainfall. These results suggest that real-time cluster detection during epidemics should be considered in Cambodia, and that improvements in weather data reporting could benefit national control programs by allow greater prioritization of limited health resources to both vulnerable populations and time periods of greatest risk. Finally, these results add to the increasing body of knowledge suggesting complex interactions between climate and dengue cases that require further targeted research.

## Background

Globally, dengue is the most widespread arbovirus, causing an estimated 390 million [95% Bayesian uncertainty interval 284–528] infections a year, of which only 96 million [67–136] are captured via surveillance systems [1]. Dengue is endemic throughout Cambodia, one of the poorest countries in Asia, which currently faces numerous health challenges with limited health infrastructure and poor economic indicators [2]. Morbidity from dengue is extensive due to a range of interconnected factors, including delays in care seeking, limited impacts of vector control, and limitations within the health sector [3,4].

Historically, large epidemics have occurred on a 3–4 year cycle, and generally all four dengue virus serotypes circulate annually; DENV-2 and DENV-3 predominated until recently, but have been largely displaced by DENV-1 since 2009 [5]. Dengue cases are reported throughout the year, but with a sharp increase during the rainy season (May to November).

A national syndromic surveillance program was established in 1980, which includes passive reporting of clinically diagnosed cases by public sector hospitals. Dengue is included in the list of the 12 priority diseases that are reported weekly to the Communicable Disease Control Department of the Ministry of Health (MOH); this reporting system contains only aggregated data on new cases and deaths for the diseases under syndromic surveillance. To supplement this system, in 2001 the National Dengue Control Program (NDCP) of the MOH implemented a hospital-based surveillance in seven paediatric hospitals or paediatric wards within referral hospitals. Weekly case reporting from these sentinel hospitals includes basic demographic indicators (age, gender, place of residence) clinical findings on admission, and status at discharge. Subsequently, this program was enhanced by the inclusion of virological and serological surveillance at five sentinel hospital sites. During the period 2002–2008, the National Dengue Control Program (NDCP) has reported on average 103 cases per 100,000 with an annual case fatality rate (CFR) ranging from 0.7 to 1.7% [3]. However, these sentinel sites are not able to capture detailed epidemiological, clinical or laboratory data, and reporting is generally incomplete; a capture-recapture study in Cambodia indicated a 4- to 29-fold underreporting rate [6]. The 2012 epidemic season was the most severe in the country since 2007, with more than 42,000 suspect or confirmed cases reported, and serves to highlight the serious epidemic potential within Cambodia.

To help address these limitations, our clinical study was designed to capture comprehensive data for a prospective series of paediatric dengue cases. The global objectives were to fully explore the epidemiology and clinical spectrum of clinical paediatric dengue in Cambodia; this portion of the study examines the captured clinical cases within the larger epidemiological context of spatial and climatic trends.

As a vector-borne disease, dengue transmission is inherently tied to weather patterns and climatic cycles, and epidemics generally peak during the wet season, beginning in the ‘hot wet’ season from May-August, and then tapering off through the ‘cold wet’ season in September. However, the fine-scale interactions of these climatic conditions on *Aedes* vector populations are poorly understood. Recent years have seen progressively larger epidemics throughout Cambodia and SE Asia; detailed spatial and epidemiological data are therefore increasingly critical to maximize the effectiveness of control programs within inherently limited budgets [7].

## Methods

### Sampling plan

Enrolment was organized weekly from September 20, 2011 to January 15, 2013 during specific surveillance days in both the internal medicine and emergency wards of the National Paediatric Hospital, Phnom Penh. All children aged 1–15 years and presenting with fever, or history of fever within seven days, on admission were screened for eligibility criteria using the 1997 and the 2009 WHO dengue case definitions [8,9]. Informed consent was obtained from parents or legal guardians for all eligible children. Demographics and clinical data on admission and during hospitalization were reported on a study-designed Case Report Form (CRF). Paired sera were collected on admission and at discharge and shipped via courier the same day for serologic (IgM Capture ELISA and haemagglutination inhibition assays) and virological (virus isolation on 2 cell lines, real time RT-PCR, and NS1 antigen detection by commercial ELISA (BioRad®)) tests for dengue and other arboviral infections (i.e., Japanese encephalitis and chikungunya) as reported previously [10]. Anonymized data were entered into an electronic password-protected database by a trained data manager, using Epi-Data 3.1 (Odense, Denmark) with range-checks; data monitoring was performed by a clinician who checked the concordance between the clinical notes, the study CRF and the electronic files; discrepancies were then corrected in both the CRF and the dataset.

### Data sources

All clinical data in this study were collected by National Paediatric Hospital staff (Phnom Penh) and all serolological and virological testing were performed at the virology department of Institut Pasteur, Phnom Penh. Temperature and humidity data were obtained for the weather station in Phnom Penh [11], and precipitation was obtained from satellite estimations [12]. Population data and population density were obtained from published reports from the Ministry of Statistics (2008) [13]. Approximately 60% of the 558 dengue-positive patients resided in the greater Phnom Penh area; 216 patients were geocoded to the *sangkat* (commune/urban village) level. National-level reporting data was provided by the National Dengue Control Program at the Ministry of Health, Phnom Penh; open source base map layers were obtained from Open Development Cambodia (March 2013), see http://www.opendevelopmentcambodia.net/maps/downloads/.

### Data analysis- regressions

Analysis of the relationship between weather events and national reported disease counts utilized Poisson regression for over-dispersed data (negative binomial), and examined the time period January 2011 to January 2013. All time-series models utilized Newey-West error corrections for autocorrelated and heteroskedastic data [14]. Explanatory factors examined were the weekly mean, median, minimum, and maximum of available weather data, including temperature, relative humidity and rainfall, plus the rainfall index defined as [(cumulative rainfall X days with rainfall)/days in reporting period] [15]. Lag periods of 1–15 weeks were examined for all variables. Optimal models were identified by Akaike information (AIC) and Bayesian information (BIC) criteria; model fit was assessed using standardized deviance residuals [16]. Stata 13.1 (College Station, Texas) was used for all analyses and all tests were two-tailed, with α = 0.05.

### Data analysis- spatial

Visualization of captured cases data utilized ArcGIS 10.1 and QGIS 1.8 ‘Lisboa.’ Detailed mapping and analysis included only patients residing within greater Phnom Penh; cases were geo-referenced to the centroid of the respective *sangkat* (commune). ‘Hot-spot’ detection used SaTScan version 9.1 [17]; spatial and spatio-temporal clusters were examined with Poisson models including the underlying population density by commune. Bernoulli models were not utilized due to the limited number of dengue-negative patients. Clusters were limited to 25% of the total population, and estimations used 999 replications for Monte Carlo inference.

### Ethics review

The study was conducted according to the ethical principles of the Declaration of Helsinki of October 2002. The Cambodian National Ethics Committee for Health Research approved the overall study protocol after institutional review (approval number 123-NECHR, 22 August 2011).

## Results

**Figure 1:**
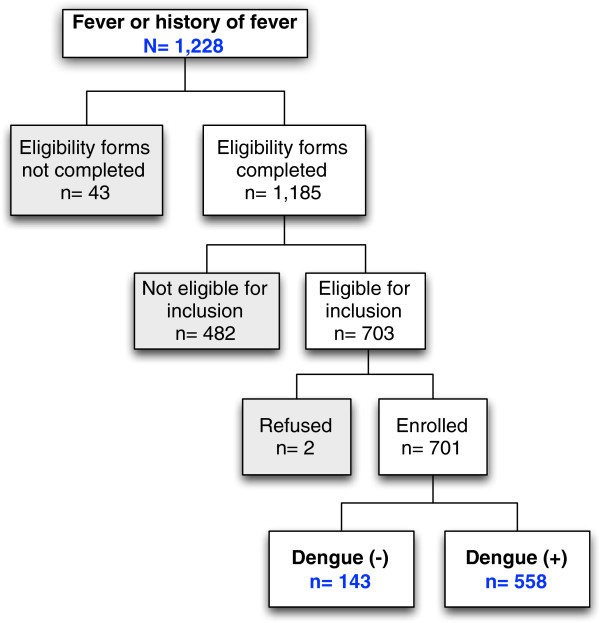
Patient inclusion flow, National Paediatric Hospital, Phnom Penh, January 2011- January 2013.

From September 20, 2011 to January 15, 2013 a total of 1,228 febrile cases were admitted to the two participating wards. An eligibility form was completed for more than 96% of these admissions (1,185/1,228); the proportion of eligible patients was 59% (703/1,185) and the study refusal rate was less than 1% (2/703). A total of 701 suspected dengue patients were enrolled during the study period, and of these 80% (558/701) had a laboratory confirmation of dengue (Figure 1).

All four serotypes were identified during the study, but DENV-1 predominated (520/558; 93%) (Table 1). Our sentinel surveillance also detected a noteworthy chikungunya outbreak reported in Cambodia in 2012: 7 patients had test results which were suggestive of recent chikungunya infection [18,19]. However, no pathogens were identified in the remaining patients.

**Table 1:** Results of the dengue serological and virological testing (N = 701), National Paediatric Hospital, Phnom Penh, September 2011 - January 2013

**Diagnosis**		**n**	**% of total**	**% of all dengue positive or negative**
			**(95% CI)**	**(95% CI)**
**Positive dengue**		558	79.6	-
			(76.4 - 82.5)	
	Dengue 1	520	-	93.2
				(90.8 – 95.1)
	Dengue 2	8	-	1.4
				(0.6 – 2.8)
	Dengue 3	1	-	0.2
				(0.005 – 1.0)
	Dengue 4	2	-	0.4
				(0.04 – 1.3)
	Serotype unspecified	27	-	4.8
				(3.2 – 7.0)
**Negative dengue**		143	20.4	-
			(17.5 – 23.6)	
	Chikungunya	7	-	4.9
				(2.0 – 9.8)

Overall the proportion of under-5 children was 18% (128/558) with an equal number of boys and girls. The onset of fever prior admission was less or equal to three days for 18% of subjects (126/558), and the median duration of hospital stay was 3 days. Overall, there were 5 deaths in the study population, indicating a case-fatality rate of 0.7% (95% CI, 0.2–1.7%).

**Figure 2:**
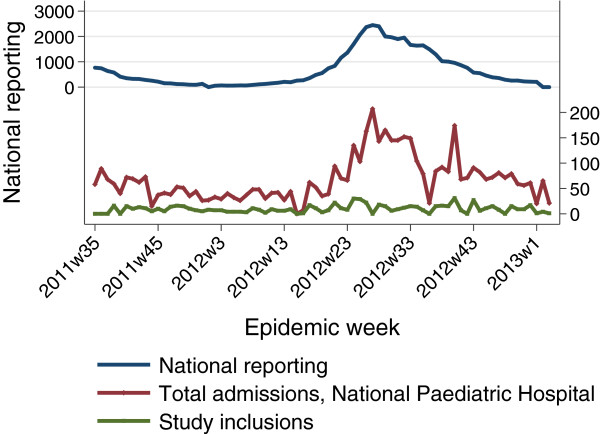
**Comparison of national reporting data versus hospital inpatient cases, National Paediatric Hospital, Phnom Penh September. 2011- January 2013.** Note: (p < 0.001, time-series regression of total hospital admissions and national cases).

**Figure 3:**
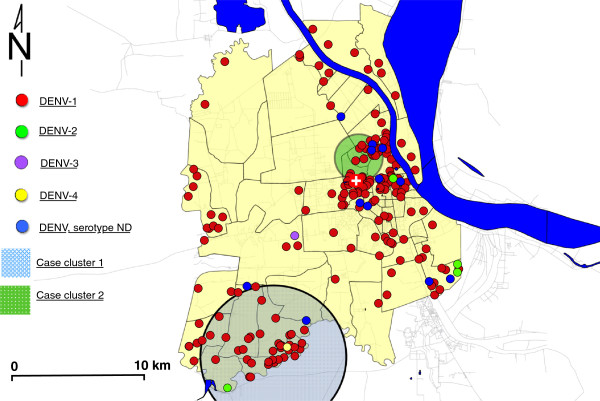
**Mapping of cases by serotype showing significant spatial clusters within greater Phnom Penh, by smallest administrative district (sangkat) (n = 216).** Note: Cluster 1 (radius- 5.2 km): 46 cases; relative Risk = 2.1 (p = 0.004); Cluster 2 (1.7 km): 43 cases; relative Risk = 1.9 (p = 0.031).

**Figure 4:**
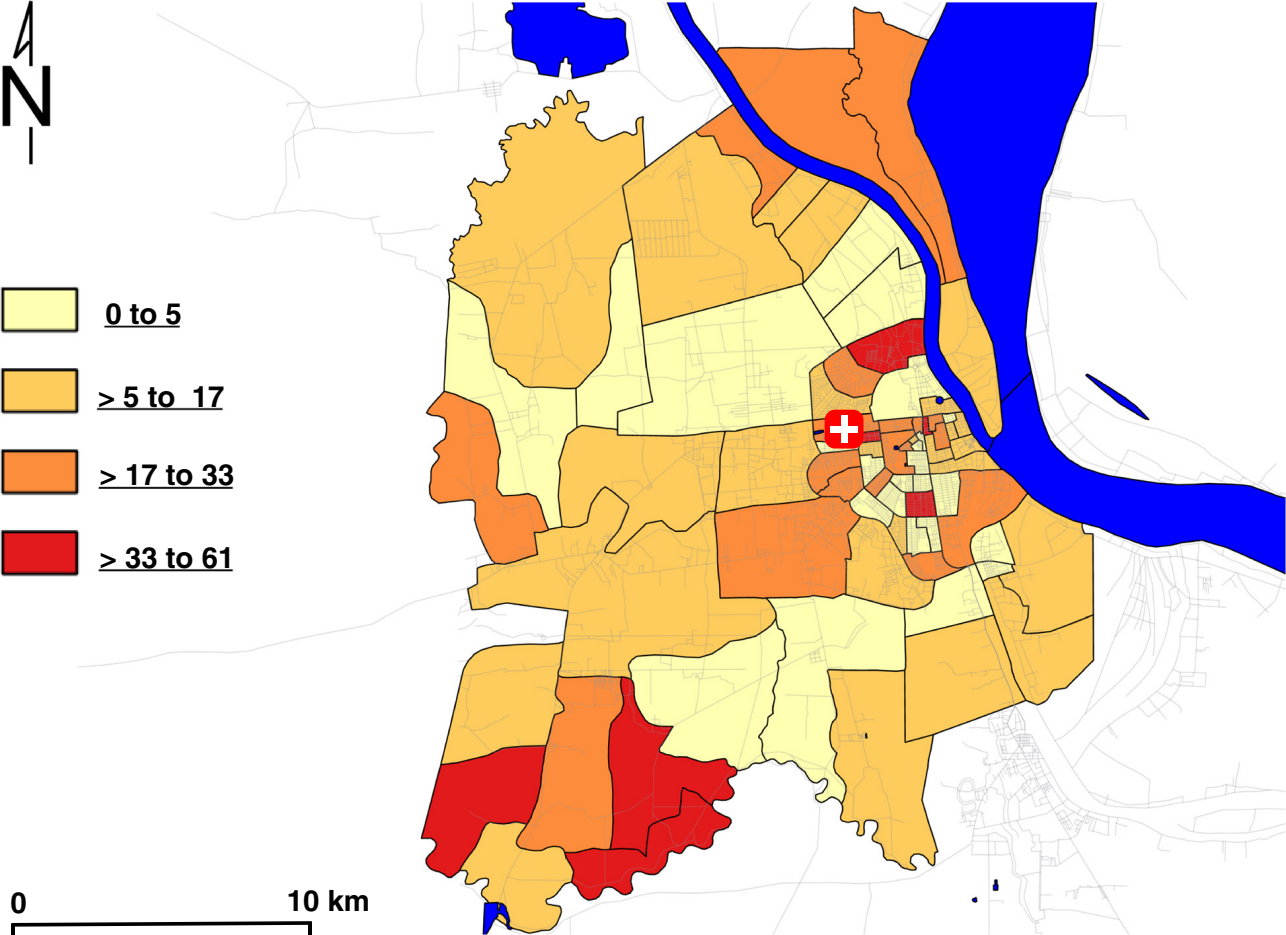
**Mapping of cases as annual population standardized rates per 100,000 total population (2008 population estimates) by smallest administrative district (*sangkat*) (n = 216).**

To assess the representativeness of our hospital cohort relative to the national epidemic, we compared the weekly caseload of admissions at the National Paediatric Hospital with the figures reported nationally (Figure 2). While this represents a crude proxy, the relationship is statistically significant across the study period when compared by simple correlation (R^2^ = 0.67; p < 0.001) and by time-series regression (p < 0.001). These results indicate that the temporal trends within the hospital and across Cambodia are highly correlated and suggest that the admission trends in cases captured in our study are indeed representative of the national-scale dengue epidemic.Two purely spatial clusters were identified (Figure 3). In the first cluster, 46 cases were aggregated within the southwest of the Phnom Penh suburban area over the entire reporting period (RR = 2.12, no CI; p = 0.004). The second cluster includes 43 cases aggregated in central Phnom Penh (RR = 1.93, no CI; p = 0.031). These p-values of less than 0.05 suggest these specific case aggregations are unlikely to be due to chance. In spatio-temporal analysis, only a single large cluster (radius > 9 km) was found to be marginally significant (p = 0.04) during October 2011 (data not shown). The standardized annual rates (per 100,000 population) within the city are shown as a choropleth map in Figure 4; highest rates are evident in the southwest and central regions, and these regions of highest risk coincide with the clusters identified in the spatial analysis. To assess any biases in capture of patients with geocoded addresses, the baseline demographics of patients were compared to the overall study population, and no significant differences were found (gender by rank-sum test, p = 0.302; age by Kruskal-Wallis test, p = 0.063).

**Figure 5:**
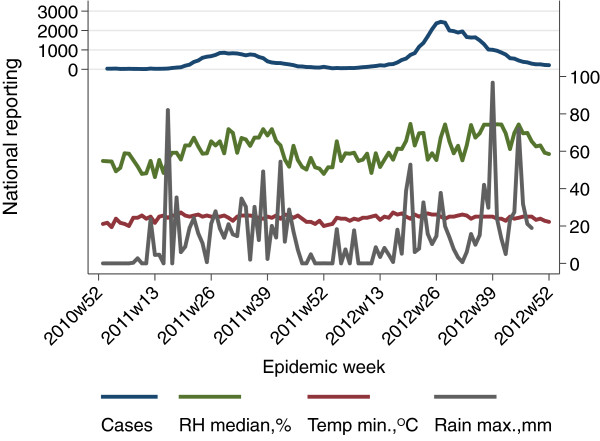
**Comparison of national reporting data and significant climatic variables, Cambodia national data, December 2010- December 2012.** Note: RH = relative humidity.

In the analysis of climatic impact on national dengue reports, three examined variables had greatest explanatory power in bivariate analysis: minimum weekly temperature; median weekly relative humidity; and weekly maximum rainfall (see Additional file 1: Table S1). The minimum temperature was positively correlated with the number of cases nationwide, with significant effects at lagged intervals of 1–3, 6, and 9–11 weeks. The corresponding incidence rate ratios (IRRs), ranging from 1.12 to 1.22, indicate a 12-22% increase in cases per degree Celsius increase in the weekly minimum temperature. The median relative humidity was also positively correlated with dengue cases in multivariate models, having significant effects at 1–3 weeks, with an IRR ranging from 1.036-1.042, indicating a 4% increase in cases for each unit increase in the weekly median relative humidity. Figure 5 illustrates the general relationships between national reported cases and significant climatic variables from multivariate analysis.

Among the examined rainfall variables, the maximum weekly rainfall showed the strongest association with reported dengue cases, with a positive correlation in bivariate analysis. However, in multivariate analysis the coefficients became negative, indicating an inverse relationship with dengue cases after adjusting for covariates. Lag periods of 1–5, and 10–12 weeks were all significant, with IRRs ranging from 0.991–0.987, indicting a 0.9–1.3% decrease in cases per mm of rain per week. Analysis of data from the other weather station in Cambodia (Siem Reap; not shown) indicates the same general multivariate trends, however these data are limited by extensive missing values.

## Discussion

We present the results of a large prospective descriptive study of paediatric dengue infection conducted in a single referral centre in Phnom Penh, Cambodia. All 701 children enrolled in the study were initially suspected as having dengue, and nearly 80% included had a positive confirmatory test. The inclusion period was from September 2011 to January 2013, which covered two dengue epidemic seasons. Although all four serotypes were found in circulation during the period, DENV-1 predominated in 2012–2013; these trends are consistent with data from the National Surveillance system [5].

Spatial epidemiology has been specifically highlighted as a key component to guide dengue control efforts, especially in resource-limited settings. A recent analysis of large-scale transmission patterns of dengue in Cambodia identified ‘hotspots’ - zones of high transmission that may serve as reservoirs of virus for subnational scale epidemiology [20]. Extensive fever cohorts have found large differences in transmission within and between urban and rural regions [21]; however, these studies did not address neighbourhood-scale effects, which are dominated by human movements between neighbourhoods. The results from this study show substantial heterogeneity of transmission within Phnom Penh, mirroring larger scale infection dynamics within Cambodia. Our results suggest that rapid identification and targeting of these areas could have important impacts on transmission, especially in areas of high population density.

When examined as total cases per population for the study period, we identified several areas of elevated risk in the southwest and central areas of Phnom Penh aggregated over the course of this study. Such clusters could be high-risk areas for dengue epidemics due to larval sites or other modifiable characteristics and should be prioritized for vector control activities including community health promotion & education, elimination of breeding sites, water treatment using larvicides, and isolation of fever cases. Recent large-scale studies conducted in Bangkok found long-term modulation of transmission based on population-level (‘herd’) immunity at the neighbourhood scale [22]; our study findings are consistent with these results. The overwhelming predominance of a single serotype, however, hindered investigation of serotype-specific clusters and any potential interactions on population immunity.

The time lags identified within this study are broadly consistent with those found in other locations within SE Asia: in Thailand lag times of 6 months for temperature maxima and 3 months for rainfall events were identified; in Singapore, lag times of 1–5 months for mean temperature and rainfall events were identified [23,24]. In common with studies in Sri Lanka and New Caledonia, we find very limited and complex impact of rainfall on dengue incidence [25,26]. A main limitation is that the rainfall data are derived from satellite imagery, and may have limited spatial and temporal accuracy. However, satellite imagery in general has been shown to be most accurate for low latitudes during the warm season [27], suggesting that these estimates are likely reasonable. Additional support for this interpretation is the finding of an inverse relationship between rainfall and *Aedes* larval densities in Malaysia, suggesting a ‘flooding-out’ of larval habitats during periods of extreme rainfall [28]. These results concur with recent studies that found complex non-linear interactions between climatic variables and dengue incidence [29].

### Limitations of this study

These data have been collected in a single referral paediatric hospital in Phnom Penh during a limited period of time, and therefore our findings may not be representative of other areas with different dengue transmission patterns, populations or population density characteristics. Cases from the spatiotemporal analyses were limited to those cases with an address that could be geo-referenced; some patients provided the name of an older administrative system that could not be readily mapped; however, this bias would have led to omission of cases This would not impact cluster detection with underlying population values as any impacts would be expected to dilute the observed effect sizes. There were no significant differences in age or gender between captured patients and those without addresses, suggesting there was no differential loss to follow-up.

There is also the potential for bias in our results due to differences in care-seeking behaviour. Patients are likely to seek care at the closest referral centre, which may partially explain the clustering of the cases in this study within Phnom Penh. However, as the hospital serves as the main paediatric referral centre within the public sector, this bias is likely minor.

We have also used a single set of weather data to predict the national scale epidemic. The local-scale interactions between climate, vectors and people - that is, the micro-epidemiology and heterogeneous exposures - cannot be captured at this scale [30].

Bearing these limitations in mind, our analysis shows a very strong correlation between observed trends and national dengue data trends as well as a strong correlation between local rainfall and temperature data and satellite estimates at the national level.

## Conclusions

From an operational standpoint, the monitoring of peak temperatures, extreme humidity and rainfall indexes could help prioritize resources to be mobilized to address any emerging transmission cycles; however, the lead times identified in these data of 1–6 and 9–10 weeks might not provide sufficient time to implement aggressive campaigns in a resource-limited setting like Cambodia. Our results also suggest that a wider range of weather summary indexes should be incorporated into models to improve model fit and prediction. Recent work in malaria also suggests that predictions may be improved by using high-resolution data instead of traditional regional and weekly summaries [31], and surveillance in Cambodia could benefit from improved weather reporting systems.

While the routine use of environmental variables for real-time dengue surveillance can be complex in real-world settings [32], the wide availability of open-source tools (QGIS, R software and SaTScan) [17,33,34] make this increasingly feasible even in resource-limited settings. These results can serve as a foundation for the implementation of robust real-time surveillance within Cambodia.

Finally, the ability to pre-plan and stockpile needed resources (control staff, *Bti*, etc.) should be considered [35], and the identification of multiple hotspots of higher risk relative to the remainder of Phnom Penh suggests areas that should targeted for enhanced surveillance and vector control efforts during future transmission seasons.

## Competing interests

JLB is a paid employee of Sanofi Pasteur; YSL was a medical advisor for Sanofi Pasteur; all other authors declare that they have no conflicts of interest.

## Authors’ contributions

JB assisted in the design of the overall clinical study; all other authors participated in conceptualizing the design and coordination/data collection of the overall clinical study. AAL and PC conceived, planned, and analysed the data for this analysis and wrote the first draft; all authors read and approved the final manuscript.

## Pre-publication history

The pre-publication history for this paper can be accessed here:

http://www.biomedcentral.com/1471-2458/14/658/prepub

## Supplementary Material

Additional file 1: Table S1 Time series negative binomial regression for reported national dengue cases, Cambodia, Dec. 2010- Dec. 2012. Click here for file
