# Supplementary material for: Spatial epidemiology and climatic predictors of paediatric dengue infections captured via sentinel site surveillance, Phnom Penh Cambodia 2011–2012
Source: BMC Public Health. 2014 Jun 28;14:658. doi: 10.1186/1471-2458-14-658 (PMC4085229; doi:10.1186/1471-2458-14-658)
Supplement: Additional file 1: Table S1 — Time series negative binomial regression for reported national dengue cases, Cambodia, Dec. 2010- Dec. 2012. [file 1471-2458-14-658-S1.pdf]

Supplemental Information

Spatial epidemiology and climatic predictors of paediatric dengue infections captured via sentinel site surveillance, Phnom Penh Cambodia 2011-2012

Appendix

variable	Lag period	IRR	95% CI	p value
Minimum temperature				
	1 week lag	1.13	1.05 - 1.20	0.00034
	2 week lag	1.12	1.05 - 1.20	0.0011
	3 week lag	1.16	1.09 - 1.23	< 0.0001
	6 week lag	1.22	1.12 - 1.32	< 0.0001
	9 week lag	1.18	1.13 - 1.23	< 0.0001
	10 week lag	1.12	1.06 - 1.19	< 0.0001
	11 week lag	1.12	1.06 - 1.18	< 0.0001
Median relative humidity				
	1 week lag	1.04	1.02 - 1.05	< 0.0001
	2 week lag	1.04	1.02 - 1.05	0.00020
	3 week lag	1.04	1.03 - 1.05	< 0.0001
Maximum rainfall				
	1 week lag	0.99	0.99 - 0.99	< 0.0001
	2 week lag	0.99	0.98 - 0.99	< 0.0001
	3 week lag	0.99	0.98 - 0.99	< 0.0001
	4 week lag	0.99	0.99 – 0.99	< 0.0001
	5 week lag	0.99	0.99 – 0.99	0.016
	10 week lag	0.99	0.99 – 0.99	0.014
	12 week lag	0.99	0.99 – 0.99	0.015

Appendix figure 1. Time series negative binomial regression for reported national dengue cases, Cambodia, Dec 2010- Dec. 2012.

Note: IRR = incidence rate ratio
